# Family physicians partnering for system change: a multiple-case study of Ontario Health Teams in development

**DOI:** 10.1186/s12913-023-10070-0

**Published:** 2023-10-17

**Authors:** Colleen Grady, Sophy Chan-Nguyen, David Mathies, Nadia Alam

**Affiliations:** 1 Centre for Studies in Primary Care, Department of Medicine, Queen’s University, 220 Bagot Street, Kingston, ON K7L 3G2 Canada; 2 Muskoka and Area Ontario Health Team, Muskoka, ON Canada; 3 Institute of Health Policy, Management and Evaluation, Dalla Lana School of Public Health, University of Toronto, Toronto, ON Canada

**Keywords:** Primary care, Integrated care, Ontario Health Teams, Family physicians

## Abstract

**Background:**

The Ontario Health Team (OHT) model is a form of integrated care that seeks to provide coordinated delivery of care to communities across Ontario, Canada. Primary care is positioned at the heart of the OHT model, yet physician participation and representation has been severely challenged at planning and governance tables. The purpose of this multiple case study is to examine (1) processes and structures to enable family physician participation in OHTs and (2) describe challenges to family physician participation.

**Methods:**

We chose a qualitative, exploratory multiple-case study approach following Yin’s design and methods. The study took place between June and December 2021.We conducted semi-structured interviews with OHT stakeholders in four communities and carried out an analysis of internal and external documents to contextualize interview findings. Thematic analysis was applied within case and between cases.

**Results:**

Four OHTs participated in this study with thirty-nine participants (17 family physicians; 22 other stakeholders). Over 60 documents were analyzed. Within-case analysis found that structures and processes should be formalized and established to facilitate physician participation. Skepticism, burnout, heavy workload, and the COVID-19 pandemic were challenges to participation. Between-case analysis found that participation varied. Face-to-face communication processes were favoured in all cases and history of collaboration facilitated relationship-building. All cases faced similar challenges to physician participation despite regional differences.

**Conclusions:**

The implementation of OHTs demonstrates that integrated care models can address critical health system issues through a collective approach. Physician participation is vital to the development of an OHT, however, recognition of their challenges (skepticism, burnout, COVID-19 pandemic) to participating must be acknowledged first. To ensure that models like OHTs thrive, physicians must be meaningfully engaged in various aspects and levels of governance and delivery.

## Background

An integrated health system seeks to decrease patient transitions, minimize deleterious impacts on health outcomes, and reduce ever-rising taxpayer costs in many countries [1, 2]. While there are multiple definitions to describe integrated care [2], the essence of each remains the same: to reduce barriers between services by improving coordination among healthcare providers and organizations. Physician perspectives can play a significant role in shaping integrated care delivery to be more efficient, safe, and patient-centred [3] due to their role in continuity of care to patients throughout the lifespan. However, communities across Canada are facing dire shortages of family physicians (FPs) [4, 5] as they find themselves increasingly burdened with administrative duties, hampering meaningful participation in system reform. There is little global literature examining physician participation meriting broader examination of the topic.

### Healthcare integration

Inspiration for past integrated care initiatives have been drawn from a variety of models including those in the United States and the United Kingdom [6]. The processes and outcomes of an integrated care model can vary significantly depending on the various stakeholders involved (physician groups, patients, policymakers, researchers, and the public). Integrated care models and outcomes also overlap with other initiatives that seek to bring about healthcare system reform through collaborative means.

Large system transformation initiatives adopting a complex adaptive systems approach also aim to create coordinated, systemwide change by involving multiple organizations and care providers [7]. Likewise, health alliances implemented in New Zealand feature and rely on specific governance arrangements across districts to support integration between different healthcare organizations [8]. Healthcare alliances feature a leadership team to ensure top-level governance and includes members from a variety of service providers and consumer groups from different districts. The desired result is to shift services from hospitals to primary care settings with the goal of cost savings [9].

Of significance, Accountable Care Organizations (ACOs), a model of integrated care originating in the United States, have shown the effectiveness of integrated care between providers. An ACOs’ primary outcome is on shared savings that are reinvested into the organization toward prevention and cost reduction [10]. The financial incentive attached to an ACO is a powerful push toward integration. Savings can then be reinvested to benefit communities [10].

In Ontario, Canada, integrated care initiatives have been underway since the early 2000s including rural health hubs, integrated funding models, Community Care Access Centres, Francophone services, and Indigenous Healing and Wellness Strategies [11]. Ontario Health Teams represent the most comprehensive approach to date, due to efforts to connect large networks of diverse providers [11].

### Ontario Health Teams

In June 2018, the Ontario government led a major shift in the healthcare system and announced that the fourteen regional health networks known as Local Health Integration Networks (LHINs) would be replaced with a localized, community-driven approach called Ontario Health Teams (OHTs) [12]. LHINs were crown corporations established through Bill 36, the Local Health System Integration Act, to create regionally-integrated health systems and allow for local planning [12, 13]. OHTs differ from LHINs as membership is drawn from the communities they serve whereas board members of previous LHINs were appointed by the government and remained accountable to the Ministry of Health. While the intent of both models of health system integration are the same – decentralization of health system management, and promotion of a seamless system of care for patients, OHTs are ‘ground-up’ and unique to each community.

Regions and communities were defined for LHINs and OHTs by patient access patterns, with LHINs covering wider geographic areas than OHTs, evident in the number of each (14 LHINs vs. 42 OHTs). Neither model provided financial incentives for local community healthcare organizations to collaborate, although OHTs represent a better opportunity for communities to work together on local priorities as they are community-driven with relationships between organizations and providers already in place.

Additionally, one super-agency, Ontario Health, would be established to provide provincial supports in alignment with the Quadruple Aim– reducing costs, improving population health, patient experience, and better provider experience [12, 14]. OHTs were designed “to work as one coordinated team to improve patient outcomes, strengthen local services, and make it easier for patients and families to navigate the system and transition between health care providers” [15].

Communities applied to become an OHT with strict criteria related to the inclusion of partners. Partners are diverse, including primary care, hospitals, community support services, and patient and community partners [15]. Steered by provincial guidelines, each OHT defined regional priorities to create a localized approach to system integration. Between December 2019 and November 2020, 42 OHTs were approved, covering 86% of the provincial population [16].

Partnerships between providers are necessary for integration. Primary care is arguably one of the critical partners within the OHT, often referred to as ‘the cornerstone’ [17, 18] in a healthcare system. FPs have intimate knowledge of service gaps, patients’ needs, and community services as they see more patients than other providers [19]. Partnering for change is not without its challenges [1]. FPs, the provider of the most-oft used type of care, experience substantial challenges to participate in integrated care initiatives [20]. FPs in Ontario are independent businesses and may subscribe to one of several practice models (solo or team-based) or payment models (fee-for-service, capitation). Payment models are products of government policy and individual choice. Some payment models, such as those attached to Family Health Organizations as opposed to Family Health Groups or fee-for-service, have greater representation in service due to the stability of the payment structure. The lack of a common structure used by FPs in most communities challenges coordination for consistent representation at health system tables and a stable mechanism for information exchange.

Emerging studies on OHTs have focused on its evolution and growth [21, 22], engagement of patient and family advisors in health system redesign [23], collaborative leadership, and early lessons [24]. However, few studies have explored the factors impacting FPs’ participation in the development and maturation of OHTs [25]. Without FPs as part of reform efforts, OHTs will struggle to foster regional change and build a transformative and sustainable integrated healthcare system [15, 26].

The purpose of this study is to explore how FPs are participating in OHTs, specifically, whether any organizing structures and/or key processes enabled them to do so. Drawing on functional organization theory, this study aims to draw out insight to solutions that increase FP participation in OHTs. Two research questions guided this project:

1. How are FPs engaging with and/or being engaged by the OHT, and were there any structures or processes that were beneficial to participation?
2. Why is it challenging to include FPs in OHT decision making and system change?

Through a multiple-case study design, this study aims to answer these questions by examining FP participation in four diverse communities, highlighting real-world successes and challenges related to system integration.

## Methods

**Fig. 1:**
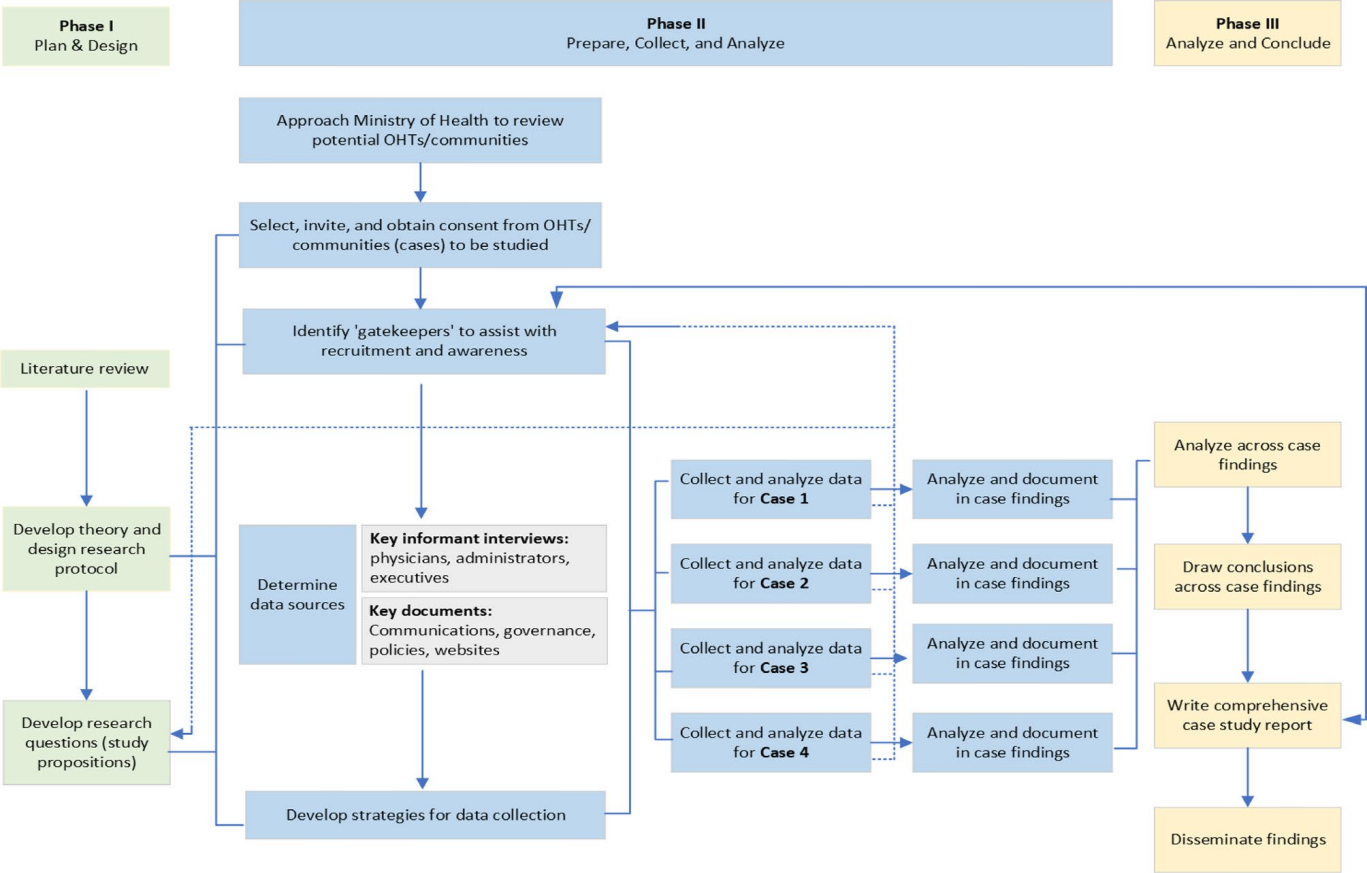
Study flow chart detailing the multiple case study design and approach, adapted from Yin & Campbell (2018) and Burrows et al. (2020)

We chose a qualitative, exploratory multiple-case study approach for this study, following Yin’s design and methods [27]. As OHTs are community-driven, a Yinian approach which allows for comparisons of similarities and differences between cases and seeks to understand “a contemporary phenomenon within its real-life context” was fitting. The case study design utilized a constructivist worldview in which insights and interpretation is drawn from the lived experiences and perspectives of the participants. Consistent with this approach, we have adhered to a systematic progression of research components to remain true to the ‘yardsticks’ identified by Yin to ensure rigour in the research. These criteria include: construct validity, internal validity, external validity, and reliability [27]. The uniqueness of each case further supported the researchers’ decision to use multiple-case study and allow for cross-case analysis and a deeper exploration of theory related to the need for structures and processes to support the participation of FPs in OHTs [28] (Fig. 1).

### Phase I: plan and design

#### Study design

A case study protocol was developed; it included the project workplan and definitions, roles and responsibilities of the project team members, a framework for data analysis, and timeline to completion. As per Yin’s approach to case study, the research questions that guided this investigation focused on the ‘how’ and ‘why’ of the phenomenon.

### Theoretical framework

Burrell and Morgan’s sociological theories, specifically the principles within the functionalist paradigm, often used for organizational study, provided the theoretical framework for this research [28]. Theories within this paradigm are based on the notion of stability and order for a healthy and productive society; structures and processes designed to support ongoing participation and decision-making by FPs are functional in nature and viewed as increasing stability.

### Phase II: recruitment, Data Collection and within-case analysis

#### Setting

This study was conducted in Ontario, Canada. Purposive sampling was used to identify cases using a table that was developed to define OHTs by their location (northern, western, etc.), their approval date (Phase 1 or 2), and type of location (rural, remote, urban). From there, cases were identified that presented maximum diversity. OHT Leads (Chair or Physician Lead) from each OHT were sent a letter of invitation by email, seeking their interest in participating in the study. Five OHTs were identified and invited; four OHTs agreed to participate.

#### Recruitment of participants

Following agreement with a lead at each of the four participating OHTs, we identified a gatekeeper for each case (usually the same Chair or Physician lead). The gatekeepers communicated to OHT partners about the study, responded to our requests for documents and provided an initial list of key informants for interviews and assisted with recruitment. In three of the four cases, gatekeepers also participated in interviews. Informed consent was obtained from all participants via email and reviewed at the beginning of each interview.

Key informants in each case included both FPs and non-physicians (administrators, healthcare partners that were OHT members) who would provide diverse perspectives on participation in OHTs, utilizing one interview guide for all. The goal for recruitment was to have a balanced mix of participants and to achieve thematic sufficiency. Purposeful and snowball sampling were both used. A thank-you gift card was provided to each participant.

#### Data collection

Data were collected from three sources: key informant interviews, archival documents (pre-OHT stakeholder presentations, press releases), and administrative documents (meeting agendas, minutes).

Prior to conducting interviews a guide was developed based on ‘how’ and ‘why’ research questions and by the theoretical proposition that some degree of organization by FPs would enable their collective participation in OHTs. The three primary areas of questions included: how FPs were participating in OHTs, why was it important for FP participation in OHTs, and why were there challenges (if any) to achieving this participation. Three authors (SCN CG, NA) conducted semi-structured interviews virtually. Of the three study team members collecting data, NA is a family physician. Prior to interviews, a group training session was held including a mock interview to ensure that investigators were using the same approach and that interview questions were clear. The interviews took place between June and November 2021 and lasted between 35 and 90 min.All interviews were audio-recorded and transcribed verbatim. In each case, interviews were shared between two investigators to provide optimal availability for participants.

The second data source was archival documents related to the four cases. This included past community network documentation (if any pre-empted the OHT), news/online stories, press releases or op-eds related to OHT development, and stakeholder consultations or presentations given prior to approval as an OHT. Prior to COVID-19, many communities promoted the OHTs and informed potential partners about the application to become an OHT. These discussions were documented and included PowerPoint presentations along with an attendee list.

The third data source included documents considered to be current and generated after approval of the OHT by the province. These included meeting minutes and agendas from steering and physician committees, and administrative documents related to the governance of the OHT (decision-making frameworks, OHT application, organizational charts).

All data (transcripts and documents) were stored in an encrypted drive accessible by three investigators.

#### Data analysis

Within-case analysis was completed by two authors (SCN, CG) using NVivo 12. Neither SCN or CG are family physicians but have substantive experience working in primary care research. Each investigator coded a transcript independently, compared coding, discussed nuances within codes to achieve consensus, and established a codebook. SCN and CG each took the lead in two cases, with one researcher completing the analysis of interview data while the other completed the document analysis for those same two cases, increasing rigour in the analysis.

SCN and CG each summarized findings for two cases and presented to one another for group interpretation and discussion. The documents were read over to contextualize the major themes found in the study. Throughout data collection and analysis phases, we consulted the gatekeepers and collected additional documents to fill in gaps in information. The final themes were presented to the rest of the study team for review and validation. Throughout the analysis of each of the four cases, copious notes were taken to document the discussions which enabled the two investigators (SCN and CG) to conduct a cross-case analysis.

A document analysis matrix was developed to allow for consistency and identified; document date and type, how obtained, content summary, and any reference that aligned with the theoretical proposition that the collective participation of FPs was evident.

After finalizing the themes, the main findings of each case were summarized into a draft report. We shared the report with each gatekeeper for verification of the findings and interpretation. This triangulation of data with the gatekeepers is consistent with Yin’s criterion of internal validity which increased our ability to capture accurate knowledge in each case [27]. Following minor revisions, the final reports were provided to gatekeepers for dissemination within their OHTs.

### Phase III: cross-case analysis and conclusions

Subsequently, we revisited the data, both within-case and across-case, and by analyzing between and across cases, we were able to understand key differences, or factors, that strengthened, or challenged, OHT development.

### Quality of research

Returning to Yin’s research design quality criteria, investigators remained mindful of each criterion throughout the study.

1. Construct validity was reinforced by drawing on multiple sources of evidence (e.g. semi-structured interviews, OHT-related documents, archival documents) and also by asking gatekeepers, who were also key informants, to review the summary of results.
2. Internal validity was reinforced using triangulation techniques with multiple investigators and two coders plus the application of pattern matching logic in the cross-case analysis, seeking whether individual cases show similar outcomes in organizing for FP participation. Rival explanations were explored when results were presented and discussed by the full study team and when stakeholder reports were provided to each gatekeeper for feedback and/or confirmation.
3. External validity has been achieved using replication logic with the multiple-case method, as each case presented enough similarities as a developing OHT to enable the inquiry related to similar structures and processes.
4. Reliability has been attended to with a well-developed research design and protocol, investigator interview training and documented processes. This allowed for consistency of process between investigators.

### Ethics approval

The study was approved by Queen’s University’s Health Sciences Ethics Review Board (HSREB), file #6032595.

## Findings

This study sought to understand how FPs are engaging with or being engaged by OHTs via a unifying structure for decision making purposes (committee, council, alliance) or by employing specific processes that enabled participation. We also wanted to understand the challenges that FPs faced in OHT decision making and system change. In total, 39 interviews were completed, 17 (44%) of those were with FPs (Table 1) and 22 (56%) were conducted with other OHT stakeholders such as executive directors of health care teams (13, 33%), hospital directors (2, 5%), managers (4, 10%), and consultants (3, 8%). We aimed to have 10 individuals from each case participate, with a total of 40 participants in total. The inclusion of non- FPs was intended to capture broader cultural or structural factors at play in FP participation and to examine whether views on FP participation differed from those of FPs.

**Table 1:** Case characteristics

**Case 1** • Cohort 2, approved Nov 2020 • Geography: Rural region • Participants: 4 FPs, 6 non-FPs	**Case 2** • Cohort 1, approved Dec 2019 • Geography: Rural/northern region • Participants: 4 FPs, 6 non-FPs
**Case 3** • Cohort 2, approved Nov 2020 • Geography: Rural/urban mix region • Participants: 5 FPs, 6 non-FPs	**Case 4** • Cohort 2, approved Nov 2020 • Geography: Rural/remote/northern region • Participants: 4 FPs, 4 non-FPs

Table 2 highlights the major themes that emerged from the cases that answers the research questions.

**Table 2:** Themes from each individual case

**How are FPs engaging with and/or being engaged by the OHT, and were there any structures or processes that were beneficial to participation?**
Case 1 • Generational differences in FP engagement (Younger FPs more likely to participate) • Physician champions influential • Process: Need for process; immature at moment • Structure: Previous primary care structure; standalone and separate from OHT
Case 2 • Generational differences in FP engagement (Younger FPs more likely to participate) • Representation from all sites • Process: ∘ FPs involved in development of governance model ∘ COVID-19 pandemic forced FP engagement with OHT • Structure: OHT model built on previous project successes
Case 3 • Physician champions influential • Process: FPs invited to table early during OHT application process; High level of FP participation • Structure: Established through early buy-in and engagement through a physician council
Case 4 • Minimal to no processes and structures in place • Some existing structures prior to OHTs influencing collaboration, but on a grassroots level
**Why is it challenging to include FPs in OHT decision making and system change?**
Case 1 • Skepticism of change • Administrative heavy/not relevant to FPs • Meetings not good investment of time • Inconvenient meeting times • Lack of compensation • Tangible outcomes needed • Lack of FPs in region • COVID-19 pandemic interrupting progress • Burnout and heavy workload • Challenged communication
Case 2 • Meaningful and consistent communication methods needed • Skepticism; promises unfulfilled previously • Government and hospital control; power imbalance • Time commitment and high workload • COVID-19 pausing most OHT activities
Case 3 • Skepticism, cynicism towards change • Silos and power imbalances between different actors • Workload and burnout • FPs volunteering • Unequal resources in region • COVID-19 pausing most OHT activities
Case 4 • Compensation model • Limited resources • No consultation • No tangible impact • Power imbalance • Skepticism and cynicism towards change • Burnout and workload

There were several shared themes among cases, and some outliers identified by within-case and cross-case analysis. Three broad themes were: (1) structure for decision-making, (2) processes related to communication and relationship-building/collaboration, and (3) challenges to FP participation (Table 3).

**Table 3:** Shared themes within cases

	Themes	Case 1	Case 2	Case 3	Case 4
**1.**	**Structure for FP participation was viewed as valuable**	Structure pending development	Well-defined structure in place and active participation	Well-defined structure in place and active participation	Discussions started related to a FP structure
**2.**	**Communication with FPs a challenge** **Relationship-building/collaboration pivotal to OHT development**	Not well, not “super-strong”, “horribly piecemeal” Collaboration between FPs evident within small communities but minimal across the region	Too much info, too little that’s relevant to FPs; consistent mechanism needed High degree of previous collaboration through similar integration work very beneficial to OHT development and FP participation	Strong start to FP communication but challenged by different practice models Power imbalances between providers and OHT partners had detrimental impact on OHT development	Communication minimal, efforts underway to create website Minimal region-wide collaboration between FPs due to remote nature of services but some situational collaborating evident
**3.**	**Challenges;** **Skepticism** **FP Workloads** **Pandemic impact**	Some skepticism from FPs re: OHT success Burnout and heavy workload of FPs seen as poorly understood by non-FP OHT partners Pandemic shifted focus away from OHT work	High degree of skepticism from FPs noted Overworked FPs had limited their ability to participate Pandemic response viewed as a success due to collaboration history	Some skepticism from FPs re: government FPs noted that an integrated care model may increase the burden on them Pandemic stalled OHT development	Minimal skepticism from FPs noted; enthusiasm instead for OHT work Workload for FPs exacerbated by need for multiple roles in rural regions Pandemic pushed back all OHT activities; late start in securing admin lead, digital presence

Outliers represent actions or outcomes which either enabled FP participation or otherwise made their participation even more challenging and were evident in some of the shared themes (Table 4).

**Table 4:** Cross-Case Analysis: exploring outliers within themes

**1.**	**Structures for decision making** A strong emphasis on establishing a governance structure for FPs was clear in 2 cases. In both, FPs were participating at governance and community levels with one making a significant effort to ensure representation on all working groups In one case FP participation was primarily at the governance level In still another case, there were no FPs participating in any role.
**2.**	**Communication successes** Face-to-face communication was highly favoured in 1 case, viewed as respectful by FPs which resulted in more participation by FPs FPs in rural communities that practiced in hospitals frequently received OHT updates from the Medical Advisory Committee which kept them informed **Relationship-building/collaboration** In 1 case, relationships that pre-existed before the OHT allowed this community to ramp up quickly as an OHT In another case, history played a detrimental role as power imbalances and challenges in partnering made engaging with FPs difficult. In one case engagement with a large First Nations community highlighted the need for cultural safety training for all partners

## Within-case analysis results

This section details the findings of the within-case analysis. Table 2 demonstrates the major themes that emerged, showcasing the similarities and differences of each case.

The following will focus on three shared themes found within each case as highlighted in Table 3.

### 1) Structure for decision making

In each case, the need to have a formalized structure for FPs (committee, council) was seen as a valuable mechanism for communication and decision-making.

*“On the primary care side, they have to be able to organize, to speak with one voice so you’re not getting six different answers from six different practices”(non-FP/Hospital Director).*

### 2) Processes

Communication strategies and relationship-building with FPs were priorities to enable FP participation.

#### Communication

Communication with FPs was a challenge in all cases. Email was the method used most often by OHT administrators for sharing information although the least preferred by busy FPs and easily overlooked *“when you get 5 billion emails all the time”(FP).* Despite a lack of time, FPs identified that, where possible, in-person communication was the best way to deliver information *“because a face-to-face meeting is worth a million times an email in your inbox”(FP).* Meetings that took place after work hours enabled FP participation as it would not take them away from their clinical work, however, after hours meetings were few and far between. Preliminary presentations were made to the primary care sector at the time of OHT approval and/or application in most cases. This was seen as a good opportunity for generating dialogue with FPs but did not continue once the OHT was approved.

#### Relationship building/collaboration

Partnerships are dependent upon relationships. In all cases, relationships played a key role in OHT development. Building trust takes time and previous collaborative attempts in a community can either amplify good working relationships or resurrect previous clashes that hinder movement. In three of the four cases FPs referred to *“some historical stuff between hospitals and community”(non-FP/Executive Director)* which impeded progress.

Building an integrated system of care requires commitment yet participation to date has been voluntary which limited the urgency to participate. FPs expressed that there was *“no impetus to actually force anybody to collaborate”* and people came to the OHT planning table *“because they think it’s the right thing to do”(FP).*

A history of collaboration (or absence of such) among FPs, and between FPs and other providers or organizations significantly influenced their participation, support for, and value of the OHT and impacted by various factors. Geographical factors could lead to “*a lot of openness to sharing resources and ideas” (non-FP/Manager)* due to shared needs and resources but also lead to longstanding divides for the same reasons. Differences in funding models led some communities and FPs to be more equipped to participate in system integration whereas others that were under-resourced felt less supported to do so. Other cases expressed concerns over “*internal politics around various physician groups” (*non-FP/Consultant*)* which has led to siloed communities. The turnover of previous system integration initiatives, like the LHINs, has also fostered a lack of trust among physicians to participate.

### 3) Challenges to FP participation

#### Skepticism

Skeptics were plentiful in all cases, primarily among FPs. Previous government initiatives limited their degree of enthusiasm for participating in OHTs. Several participants described their hesitation as *“having been at this rodeo before”* leading them to wonder *“is this just the repackaged, newest flavor from the government?”(FP)*. This was particularly evident among those that had been in practice long enough to see several government-funded initiatives come and go with little to show for them, diagnosing it as the *“LHIN hangover”(FP)*.

#### Burnout and workload

High levels of burnout among family physicians are evident everywhere. FPs expressed that *“we’re asked to do everything by everybody for everyone”(FP)*. Most FPs are already working extremely long days which extend into weekends and face multiple workplace stressors, severely limiting their capacity to take on more. FPs identified that non-FP members of the OHT were limited in their understanding of a FP’s workday, evidenced by *“constantly having meetings at 2 p.m. in the middle of the day: you’ll never get doctors that come out”(FP).* Workload was also exacerbated in rural regions due to multiple roles as *“they have emergency room shifts to cover for a week and then they’re doing inpatient care for their patients that are in hospital and then they’re still running a primary care practice”* (non-FP/Executive Director).

#### COVID-19 pandemic

The need to shift priorities from March 2020 and beyond for everyone in healthcare was enormous, non-stop, and stressful, and OHT development stalled in all cases. *“It’s unfortunate the way the timing of all of this played out because there was some initial momentum and then things were shut right down” (non-FP/Manager)*.

Despite having to pivot away from OHT development to deal with the pandemic, the shift for FPs to work together to operate vaccination clinics and the continual updates about changes in public health policy required them to work more closely with one another and was seen as beneficial in building relationships. FPs that previously had rarely communicated with one another were now on group zoom calls frequently.

*“It’s really enabled us to have a strong and robust COVID response. So a really integrated approach initially with the assessment centres between the Family Health Team staff and the primary care physicians doing the bulk of the testing. That kind of evolved into just being ready and able to work collaboratively by the time that the vaccine started coming”* (non-FP/Executive Director).

## Cross-case analysis results

This section details the findings of the cross-case analysis that was completed following the within-case analysis. We reviewed the themes and found some outliers or examples of unique approaches that significantly impacted OHT development.

### 1) Structures for decision-making

Although each case acknowledged the value of a structure to enable collective action by FPs, varying levels of effort were invested into establishing this structure. Two of the cases were much further advanced in this journey. In both cases, an existing or pre-existing committee was strengthened, primarily in relation to OHT activities. Committees had terms of reference, transparent governance structures, and decision-making frameworks that provided for fair representation among all FPs in the region regardless of practice model. Meetings were held with regular frequency and consistently high attendance noted. In one of these cases, a worthy effort was also made to ensure the inclusion of FPs as co-leads on all project-specific groups which increased connection between decision-making and action.

In the other two cases, the dialogue about resurrecting a previous regional planning table to facilitate family physician’s collective decision making was underway. In one, a distinct clarification about the purpose of the newly formed group structure was made to emphasize that a physician-centric committee would work “*alongside the OHT but not under the OHT”(FP)* and FPs made it clear that *“there are other issues and conversations that would benefit from collective dialogue via such a structure beyond OHT-specific items*”(FP). In the other case, there was no evidence of a previous structural model to build upon and, while viewed by OHT leads as valuable, this was largely premised on perceived expectations of the ministry, with no evidence that FPs were yet engaged in planning. Nebulous expectations from the province frustrated leadership *“It’s usually easier to reengineer a system by collaborative work and then some sort of direction. There’s very little direction here”* (non-FP/Consultant).

### 2) Processes

#### Communication

The assessment of communication strategies varied considerably between FPs and non-FPs. This was particularly evident in one case where, according to one FP, communication with OHT leads was “*not ‘super-strong”* and “*horribly piecemeal”* while we heard from non-FPs that *“we are well connected with our physicians*” (non-FP/Executive Director).

In another case, FPs acknowledged occasional face-to-face meetings with an OHT physician leader which went a long way to engaging community FPs in OHT development. This reflection in a different case by a FP illustrated just how valuable in-person communication was.

“*I think in a perfect world someone would sit down with us in a face-to-face meeting and explain to us what this OHT is going to look like and how the vision is of how primary care is going to be involved. I don’t feel like that’s happened”* (FP).

In one of the cases there was a noted failure to meaningfully engage physicians in consultation around the OHT:

*“That’s been the feel, on the ground, is the opportunity for consultation has largely been survey-based, passive, do this survey for 20 minutes and give us your opinion. As a primary care practitioner, the starting point needed to be consultation on the ground …before any bigger conversation happened about who we hire as admin support and which communities we include”* (FP).

#### Relationship-building/collaboration

In two cases OHTs were led, or co-led by a competent, respected FP with pre-existing relationships in the community. This was viewed as a key factor for success in each of these two cases. One FP noted, *“there really are the key physicians who have done a lot of the work up front, but then they can pull in other physicians in the offices*” (FP).

In one case, inequities between OHT partners and perceived power imbalances challenged progress. *“Family physicians need to be treated fairly as equal partners”(FP)* Distrust among some community partners prevented some FPs from wanting to participate; the existence of a power struggle was clear. We heard that “*it’s the specialists and the surgeons that are seen as the top ranked physicians and the family physicians and practices are local yokels… a flip-flop that needs to happen where the specialists and the surgeons are supporting the family physicians because that’s where people want to be”(FP).*

The history of collaboration varied amongst the cases. Only one case reported a strong history of collaboration with previous attempts at system integration. As a result, this facilitated a smoother transition into the OHT model, “*because of its preliminary work which started, […] that was very similar to an OHT, where there was strong collaboration” (non-FP/Executive Director).*

The rest of the cases were characterized by sporadic, grassroots-led instances of collaboration. Prior to OHT formation, two cases noted strong grassroots, spontaneous collaboration between some FPs, organizations, or communities. However, a significant divide was found between other FPs in the same cases due to differences in resources, funding models, and hospital presence. This has led to low incentive in collaboration and minimal trust amongst FPs.

## Discussion

Integrated care initiatives like OHTs have tremendous potential to positively impact patient outcomes and enact much-needed change. Primary care is central to any health care system. This study sought to fill a knowledge gap around the structures and processes used to enable to FP participation in OHTs and allow them to function as a collective group. Given that the study was carried out when most of the OHTs were in preliminary stages, relatively few structures were established. Participation of FPs is also influenced by historical and geographical factors within each region.

Having a formal structure in place to facilitate communication and decision-making by independent family physicians undoubtably enables their participation. Similarly, designing and utilizing clear and transparent processes, such as communication strategies and relationship development appear to be correlated with increased FP interest, engagement, and participation in OHTs. However, formal structures and consistent processes are insufficient mechanisms to situate primary care in the centre of integration as pre-existing barriers must be acknowledged and minimized if change is to take place. This multiple-case study not only highlights some early wins among the four cases that can be replicated in other OHTs, but also identifies some glaring hurdles that block progress.

The aim of this inquiry was to inform leadership at OHT tables across the province about challenges, successes, structural supports, and processes that enable FPs to have a viable voice in local system transformation. While each region throughout the province differs in its complement of healthcare services, capacity to collaborate, history of collaboration among providers, geography, and priority populations lessons that can be drawn from the strengths, weaknesses and challenges of each community are informative.

### Establishing structure for collective action

Even though primary care remains the largest stakeholder in any health system, unless a formalized and democratic structure is in place to support bi-directional and timely communication, the wealth of expertise among FPs cannot inform system change. The lack of a how-to guide from the province slowed down each region’s ability to ramp up quickly as a considerable amount of initial energy went into getting established [25]. Clear and distinct provincial supports and direction, minimizing the considerable start-up resources invested in determining governance, developing budget, hiring administrative staff, and planning would have been helpful. Start-up activities that are primarily focused on operations are generally of limited interest to FPs as it doesn’t make good use of their time. The scarcity of resources, training, and supports offered by the government also impacted the OHT’s ability to generate solutions in a timely fashion. Barriers that existed prior to OHTs were not removed causing substantial delays in making progress.

Partner members in an OHT must consider that the primary care sector is unique and unable to match large member organizations (such as hospitals) in freeing up representatives for mid-day meetings. Thus, authentic efforts at consultation prior to the start of OHT initiatives and throughout its development is essential. The ‘ask’ for participation must be clearly laid out for physicians. Respect for FP expertise must be demonstrated and acknowledgement of their time with fair compensation is crucial. Along the same lines, OHTs must be wary and reflexive about the possibilities of tokenism by having physician representatives in leadership or committee positions but without having engaged the wider physician population. Mechanisms such as a communication feedback loop, frequent check-ins, and reports could help physicians who have participated in OHT initiatives garner a sense of how their engagement influenced practical change.

### Enhancing communication and relationship-building processes

#### Developing communication mechanisms and leveraging post-COVID communication structures

Email can be an ineffective way to receive information, particularly if it is used as the dominant communication modality and particularly for FPs as it takes time away from patient care. OHTs could benefit from installing mechanisms that allow for two-way communication to remove power differentials by ensuring that FPs have the same degree of knowledge, time, and opportunity needed to participate in decision making as do administrators and executives. Such mechanisms would encourage dialogue and transparency. Encouraging face-to-face communication (such as personal visit from OHT Lead) could foster reciprocal communication. Admittedly, where large distances between providers limited the ability to meet in person, face to face communication is prohibitive; possibly using virtual methods of communication would be of value here. OHTs could also benefit from having consistent modes of disseminating information so that FPs can anticipate being well-informed in a systematic manner. Various communication tools and mediums should be utilized to ensure a wider audience is reached. Good communication mechanisms can serve to eliminate silos between physicians and to build trust between FPs and their OHTs.

The significant disruption caused by the COVID-19 pandemic could not have been foreseen and continues to impact the healthcare system in a multitude of ways. Whereas some indication of improved connections within communities has led to a more solidified approach to specific aspects of healthcare (i.e. mass vaccination clinics), the opportunity to retain these strengthened ties should be capitalized upon. As the pandemic wanes, the focus on OHT work will increase, allowing leadership to take advantage of any pandemic-associated communication channels or relationship building that can aid in progression of integration. Having a risk management plan in place may help to better mobilize should an adverse event (such as COVID-19) present. This is particularly pertinent given the increasing shortage of FPs in Canada affecting the growth of waitlists and the loss of care for many Canadians [4, 29].

#### Partnering for the common good

Smoother transitions between services which arise from the ability of providers and organizations to collaborate and remove operational barriers is beneficial to patients [2]. Improved integration lessens wait times, avoids unnecessary hospital admissions, and provides better patient care for the patient. The development of sustainable partnerships between providers and organizations takes time, trust, and energy. Previous issues among team members can re-appear as obstacles and prevent progress. As seen in some cases, presumed hierarchies among healthcare organizations or competition between communities limits the ability for community partnerships; on the other hand, previous community-building efforts in one case increased the ability of the OHT to quickly ramp up.

### Addressing challenges to FP participation

#### On burnout and workload: Recognition via remuneration, accessible meeting times, incentives, and evidence of success

As FPs grapple with increasing burnout as well as continued high demands on them with current crisis in family medicine [30], OHT leaders must think creatively in order to gain the participation and input from primary care. Acknowledgement and understanding of current overwhelming workloads of a FP is a good start to building relationships with this sector. Solutions are less identifiable, nevertheless, efforts to increase awareness of the limitations for FPs in participating in integration initiatives must be made. At the least, solutions should ensure that participation does not lead to an increase in the administrative burden placed on FPs.

Project managers, coordinators, and administrators could play an integral role in a physician’s workflow. Remuneration is a minimum and mandatory consideration in any request for physician participation which was evident in our study. Family physicians are busy professionals and must see that their expertise, and time, will be well utilized.

The lack of tangible incentives for the community further drains the energy from OHT work and often resembles some of the previous administration’s gains with LHINs and, seemingly, easily dismissed. Initiatives that would benefit from practical, action-based expertise must include FPs, but creative solutions are required to seek out that expertise. Reigniting strong partnerships and putting in place clear communication mechanisms could provide physicians with a clear sense of tangible outcomes for both patient care and physician working conditions. This can help physicians feel that their contributions are worthwhile.

#### On skepticism: necessity of transparency

The palpable level of skepticism among FPs will continue to limit their participation in regional initiatives unless OHT gains are evident early and relevant to family practice and patient care. Political willingness to stay the course with ongoing and increased supports is also vital as change must be seen on the ground and not simply aspirational or a project that is short-lived and can easily become extinct like the LHINs. The ability of any community to partner for change depends upon trust that all parties are working toward the same end goal [31]. The installation of meaningful and relevant performance metrics and outcomes so that challenges or shortfalls can be recognized and mitigated is necessary.

Transparency at the steering committee level and efforts to build trust among partners is necessary and formative. Simple, practical suggestions could increase transparency. Meeting minutes could be made public for FPs to read and easily access. FPs could be invited at any point in time to join a meeting as a guest and primary care leads can engage FPs early and often. The need to address skepticism is paramount as it will not only affect current attempts at rallying family physician participation but also attempts at ensuring participation in the future.

### Strengths and limitations

Our study had several strengths: the use of case study which allows for an exploration of the numerous dimensions of a developing OHT, the inclusion of multiple and diverse cases, and a team of researchers with practice expertise as a FP, and seasoned researchers knowledgeable about integrated care systems. The research design allowed for an in-depth exploration within cases and the detection of similarities and differences between cases in each unique context. Triangulation of data using gatekeepers’ knowledge and their review of preliminary reports led to increased reliability of findings and is also seen as a strength of this study.

Limitations include the capacity of the research method as it relates to generalization of findings, however, the use of multiple cases increases the applicability of findings to other developing OHTs with similar geography or at similar stages in development. The use of snowball sampling, and reliance on gatekeepers to identify potential participants naturally led to the inclusion of most FPs with some association with an OHT which may represent a biased sample. Furthermore, the goal of having 50% of participants who are FPs was not achieved with the final representation at 44%. Inclusion of FPs that were not already part of the OHT would have provided a broader perspective on FPs awareness, support for, and intention of participating in OHTs. Additionally, the presence of researchers in qualitative data collection may have impacted subject’s responses as well.

In face of mounting healthcare costs, wait-times, and increasingly complicated care demands, integrated care approaches show promise to meet longstanding challenges. OHTs present another opportunity to advance system transformation withparticipants fully supportive of the integral role of FPs to system change. While efforts are being made, risks remain for providers and organizations which are not ameliorated by a government decree to work better together. Regardless of the acknowledged necessity and value of including primary care in integration efforts, wishing it to be so is foolhardy without regard for the challenges that make it difficult for FPs to fully participate in healthcare reform. Existing skepticism must be acknowledged; a focus on fostering improved relationships with FPs through improved communications and recognition of work overload could be helpful. Making best use of a FPs time and expertise, including through formal structure which can allow for democratic process and regular dialogue which FPs can contribute to is also identified as a positive step in addressing the challenges for FPs to participate in system change. Despite the dearth of academic literature on this topic, challenges to FP participation were mostly pre-existing and well-known by nearly all participants in the study. The value of this study is found in the successes uncovered in various parts of the province which can inform and guide other integration initiatives. While more work must be done towards authentic FP participation in change efforts, Ontario is on the right path by situating the importance of primary care at the centre of the OHT.

## Data Availability

The datasets generated and/or analysed during the current study are not publicly available due to the possibility and risk of participant identification but are available from the corresponding author on reasonable request.

## Abbreviations

OHT: Ontario Health Team
FP: Family Physician
ACO: Accountable Care Organization
LHIN: Local Health Integrated Network
